# Neuroprotective Effects of Cranberry Juice Treatment in a Rat Model of Parkinson’s Disease

**DOI:** 10.3390/nu14102014

**Published:** 2022-05-11

**Authors:** Łukasz Witucki, Monika Kurpik, Hieronim Jakubowski, Michał Szulc, Przemysław Łukasz Mikołajczak, Jadwiga Jodynis-Liebert, Małgorzata Kujawska

**Affiliations:** 1 Department of Biochemistry and Biotechnology, Poznań University of Life Sciences, Dojazd 11, 60-632 Poznań, Poland; lukwitucki@gmail.com (Ł.W.); jakubows@rutgers.edu (H.J.); 2 Department of Toxicology, Poznań University of Medical Sciences, Dojazd 30, 60-631 Poznań, Poland; m.kurpik@wp.pl (M.K.); liebert@ump.edu.pl (J.J.-L.); 3 Department of Microbiology, Biochemistry and Molecular Genetics, Rutgers-New Jersey Medical School, International Center for Public Health, 225 Warren Street, Newark, NJ 07103, USA; 4 Department of Pharmacology, Poznań University of Medical Sciences, Rokietnicka 3, 60-806 Poznań, Poland; mszulc@ump.edu.pl (M.S.); przemmik@ump.edu.pl (P.Ł.M.)

**Keywords:** Parkinson’s disease, α-synuclein, apoptosis, postural instability

## Abstract

Rich in polyphenols, cranberry juice (CJ) with high antioxidant activity is believed to contribute to various health benefits. However, our knowledge of the neuroprotective potential of cranberries is limited. Previously, we have demonstrated that CJ treatment controls oxidative stress in several organs, with the most evident effect in the brain. In this study, we examined the capability of CJ for protection against Parkinson’s disease (PD) in a rotenone (ROT) rat model. Wistar rats were administered with CJ in a dose of 500 mg/kg b.w./day (*i.g.*) and subcutaneously injected with ROT (1.3 mg/kg b.w./day). The experiment lasted 45 days, including 10 days pre-treatment with CJ and 35 days combined treatment with CJ and ROT. We quantified the expression of α-synuclein and apoptosis markers in the midbrain, performed microscopic examination, and assessed postural instability to evaluate the CJ neuroprotective effect. Our results indicate that the juice treatment provided neuroprotection, as evidenced by declined α-synuclein accumulation, Bax and cleaved/active caspase-9 expression, and normalized cytochrome c level that was accompanied by the enhancement of neuronal activity survival and improved postural instability. Importantly, we also found that long-term administration of CJ alone in a relatively high dose may exert a deleterious effect on cell survival in the midbrain.

## 1. Introduction

Parkinson’s disease (PD), after Alzheimer’s disease (AD), is the second most common human neurodegenerative disorder characterized by motor dysfunction associated with a loss of dopaminergic (DAergic) neurons in the substantia nigra pars compacta (SNpc) and the formation of Lewy bodies (LBs), predominantly composed of aggregated α-synuclein [1]. About 90–95% of diagnosed PD cases are sporadic [2] and result from a combination of aging, environmental factors, and genetic susceptibility. The latter is believed to be the predominant risk factor. The pathology of the disease is very complex. Nevertheless, several underlying pathophysiological mechanisms, such as oxidative stress, mitochondrial dysfunction, and protein aggregation, which are tightly linked to impaired autophagy and apoptosis, have been credited as significant pathways for the development of experimental therapeutic approaches [3,4].

Mitochondria are cellular energy producers and maintain homeostasis in cells. Their impairment causes oxidative stress and triggers death signal pathways leading to neurodegeneration [5]. Rats exposed to long-term in a low-dose ROT treatment develop PD-like neurodegeneration due to sustained inhibition of complex I, related oxidative injury, α-synuclein aggregation, and a loss of DAergic neurons in the SNpc [6,7].

Phytochemicals modulate mitochondrial functions, apoptosis signaling, electron transport chain activity, ATP synthesis, release of reactive oxygen species (ROS), mitochondrial biogenesis, and mitophagy, thereby exerting a neuroprotective effect [8]. Cranberries rich in polyphenols (PPs) with strong antioxidant activity are believed to contribute to a wide range of health benefits. Cranberry fruits contain a high amount of PPs, such as anthocyanins and flavan-3-ols (F3O), which occur in the form of monomers (catechins) or, more commonly, as oligomers or polymers-condensed tannins (proanthocyanidins, PACs), which are distinguished into procyanidins, prodelphinidins and propelargonidins depending on the monomeric unit [9]. Epidemiological and dietary intervention studies have revealed that the intake of F3O-rich foods positively correlates with a slower cognitive decline [10,11,12] and improvement of cognitive functions [13,14,15]. PACs, besides cranberry, occur in other foods, including cocoa, grapes, apples, strawberries, red wine, and green tea [11]. Several publications have appeared in recent years documenting the neuroprotective activity of different preparations containing these condensed tannins. Strathearn et al. (2014) have demonstrated that grape seed extracts increased the survival of a DAergic cell line treated with rotenone (ROT) and rescued ROT-induced defects in mitochondrial respiration [16]. These findings support the idea that PAC-rich products may alleviate neurodegeneration in PD via enhancing mitochondrial activity [16]. Intervention studies have demonstrated improved cognitive performance in healthy older adults after dietary supplementation with procyanidin-rich pine bark extracts [17,18] and young, healthy female adults after treatment with F3O-rich cocoa [19]. Grape-derived PCAs were demonstrated to be capable of attenuating cognitive deterioration and reducing brain neurodegeneration in AD animal models [20]. Procyanidin B3, the dimer of (+)-catechin, easily crossing the blood–brain barrier (BBB), protected against amyloid-β (Aβ)-induced neurotoxicity by inhibition of Aβ aggregation and Aβ-induced intracellular ROS generation [21].

To date, however, our knowledge on neuroprotective potential of cranberry is very sparse and limited to only a few studies [22,23,24]. Anthocyanin-enriched cranberry extract has been demonstrated to exert antioxidant, anti-β-amyloid aggregation, and antiapoptotic effects in BV-2 microglia [22]. CJ has been reported to possess inhibitory activity on enzymes involved in neurotransmitters metabolism, including monoamine oxidase A, tyrosinase, and acetylcholinesterase [23]. In aging rats, cranberry PPs have been shown to improve muscle tone, strength, and balance, accompanied by enhanced neuronal functioning and the brain’s ability to respond to stress [23]. Recently, we have shown that cranberry juice treatment controlled oxidative stress in several organs, with the most notable effect in the brain [25]. This study aimed to extend the research by examining cranberry juice’s capability to modulate the apoptosis mechanism and thereby provide neuroprotection in a rat model of parkinsonism induced by ROT. 

## 2. Materials and Methods

### 2.1. Animals

In the animal experiment, we used six-week-old male albino Wistar rats (250–300 g), which were bred at the Toxicology Department of the Poznań University of Medical Sciences (Poznań, Poland). The rats were kept in groups of up to four rats per cage (Techniplast, Varese, Italy) with wood shavings in a room with a 12/12-h light/dark cycle, under temperature- (23 °C), humidity-, and air circulation- controlled conditions. The animals were provided with a commercial diet (ISO 20,000 certified laboratory feed Labofeed H) and drinking water ad libitum. 

### 2.2. Experimental Design

Commercial 6-fold concentrated cranberry juice (CJ) by Alter Medica (Żywiec, Poland), which was characterized previously in our laboratory [25], has been used in this experiment. Cranberry juice (CJ) was given to rats intragastrically (*i.g.*) at a dose of 500 mg/kg body weight (b.w.)/day. Beginning from the 11th day, animals were injected once daily for thirty-five days with ROT to induce PD. ROT (Sigma-Aldrich, Poznań, Poland) dissolved in helianthi oleum raffinatum (FAGRON a.s., Olomouc, Czech Republic) was administered subcutaneously at a dose of 1.3 mg/kg b.w./day [25]. Forty-four rats were divided randomly into four groups, with 11 animals in each, as depicted in Table 1. 

The experiment lasted forty-five days, consisting of ten days of pre-treatment with CJ and thirty-five days of combined treatment with CJ and ROT. Twenty-four hours after the last treatment, the animals were anesthetized with ketamine/xylazine (100 U/7.5 mg/kg b.w., intraperitoneally), and blood was withdrawn from the heart. The brain was quickly removed after intracardiac perfusion with isotonic sodium chloride solution. The midbrain was dissected on ice, then snap-frozen with dry ice and stored at −80 °C until further use. For the microscopic examination, the brains of three rats from each group were harvested after intracardial perfusion with isotonic sodium chloride solution, followed by 4% (*w*/*v*) paraformaldehyde in a 0.1 M sodium phosphate buffer, pH = 7.4 (Merck, Warszawa, Poland). The collected brains were fixed for twenty-four hours in the buffered 4% paraformaldehyde at 4 °C with gentle shaking. Then, the brains were subjected to subsequent exchanging with graded ethanol three times per day for three consecutive days at 4 °C.

### 2.3. Postural Instability Test

**Figure 1:**
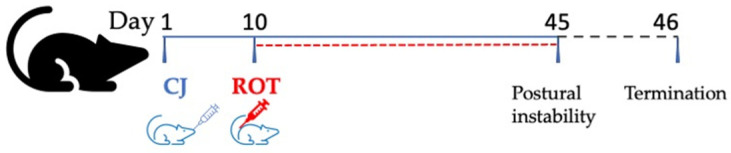
A scheme of experimental design.

This test was performed between 11:00 and 15:00, 24 h before the termination of the experiment (Figure 1), in a behavioral testing facility as previously described [7]. Each animal was held upright, and one of its forelegs was allowed to contact the sandpaper-lined table. Then, the rat’s center of gravity was shifted forward until the rat took a step. We measured the distance taken by the rat to regain its center of gravity. The average distance was calculated and reported based on three trials for each forelimb.

### 2.4. Histopathological Analysis 

Microscopic examinations were carried out in the INFO-PAT laboratory (Poznań, Poland). After the fixation described in Section 2.2, the brains were embedded in paraffin, cut into 4 μm coronal sections, and stained with hematoxylin and eosin (H&E) at 22–24 °C. The slides were examined by light microscopy (BX61VS, Olympus, Tokyo, Japan). Histopathological images were acquired with a digital camera (HVF22CL 3CCD, Hitachi, Tokyo, Japan) using the Panoramic Viewer software (3DHISTECH, Budapest, Hungary). Quantification was performed manually by counting all cells in two sections from 3 animals from each group in an unbiased way using the ImageJ software [26].

### 2.5. Western Blotting

Western blot analysis was performed to determine the α-synuclein, Bax, cytochrome c, procaspase-9, and cleaved caspase-9 protein levels. The samples prepared according to the procedure previously described [7] containing 5 µg of proteins were separated on 10% or 12% SDS-PAGE gels and transferred to nitrocellulose membranes. After blocking with 5% skimmed milk, the proteins were probed with rabbit α-synuclein (CST #4179), cytochrome c (CST #11940), Bax (CST #2772), pro-caspase-9/cleaved caspase-9 (abcam ab84786) and GAPDH (CST #5174) antibodies. All antibodies were diluted 1:1000. The Western blotting detection system and SDS-PAGE Gels (10%, 12%) were purchased from Bio-Rad Laboratories (Hercules, CA, USA). The HRP-linked antibody (CST #7074) was used as the secondary antibody. The GAPDH protein was used as an internal control. The amount of immunoreactive product in each lane was determined by densitometric scanning using a BioRad GS710 Image Densitometer (BioRad Laboratories, Hercules, CA, USA). The values were calculated as relative absorbance units (RQ) per mg protein.

## 3. Results

### 3.1. α-Synuclein Expression

**Figure 2:**
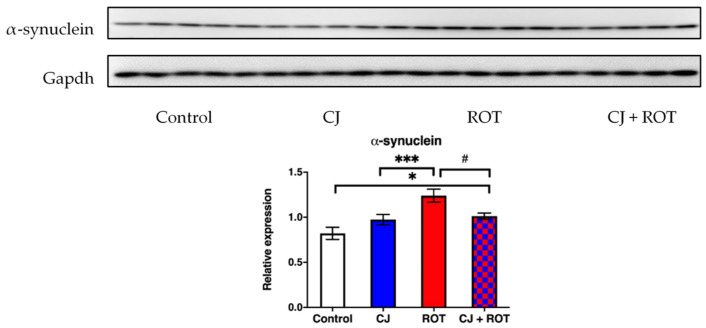
Effect of cranberry juice (CJ) treatment on α-synuclein expression. The top panel shows representative immunoblots. The graph shows the relative expression of α-synuclein normalized to GAPDH ± SEM in the midbrain of rotenone (ROT) injected rats (n = 8/group). Data were analyzed using one-way ANOVA followed by Fischer’s LSD multiple comparisons test. * *p* < 0.05 vs. Control; *** *p* < 0.001 vs. Control; # *p* < 0.05 vs. ROT.

Western blot analysis revealed that intoxication with ROT caused a 51% elevation in the level of α-synuclein compared to that of control rats. CJ administration to the ROT-challenged animals attenuated the accumulation by 18% and restored α-synuclein level near to the control level in the CJ + ROT group (Figure 2).

### 3.2. Apoptosis Markers

**Figure 3:**
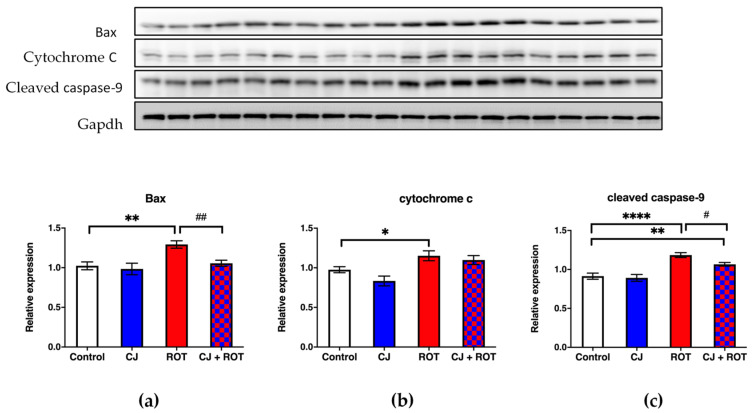
Effect of cranberry juice (CJ) treatment on apoptosis markers. Top panel shows a representative immunoblots. Graphs show relative expression ± SEM of Bax (**a**), cytochrome c (**b**), and caspase-9 (**c**), normalized to GAPDH, in the midbrain of rotenone (ROT) injected rats (n = 8/group). Data were analyzed using one-way ANOVA followed by Fischer’s LSD multiple comparisons test. * *p* < 0.05 vs. control; ** *p* < 0.01 vs. control; **** *p* < 0.0001 vs. control; # *p* < 0.05 vs. ROT; ## *p* < 0.01 vs. ROT.

ROT administration caused an increase in the expression of pro-apoptotic Bax, cytochrome c and cleaved caspase-9 by 20%, 18%, and 30%, respectively, as compared with the control values. The CJ treatment provided neuroprotection as evidenced by decreased expression of Bax and cleaved caspase-9 by 31% and 10%, respectively, and normalization of cytochrome c level (Figure 3).

### 3.3. Histopathological Analysis

**Figure 4:**
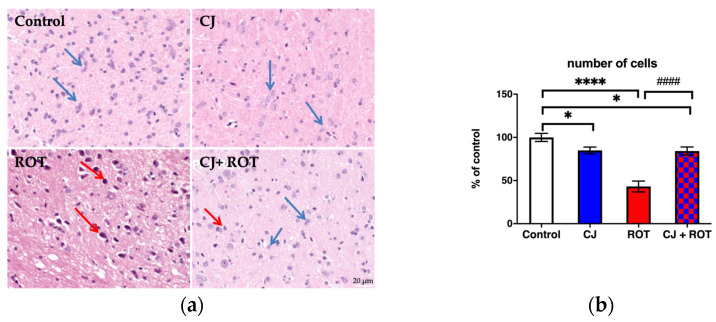
Representative photomicrographs of rat midbrain sections stained with hematoxylin and eosin (H&E). Normal neurons (blue arrows) are seen in Control and CJ alone treated rats. ROT administration caused noticeable degeneration of neurons (red arrows). Rats treated with CJ + ROT exhibit normal neurons (blue arrows) with a few cells with signs of degeneration (red arrows). Scale bar—20 μm (**a**); Quantification of cells on two sections from 3 animals from each group expressed as a percent of control (**b**). Data were analyzed using one-way ANOVA followed by Fischer’s LSD multiple comparisons test. * *p* < 0.05 vs. control; **** *p* < 0.0001 vs. control; #### *p* < 0.0001 vs. ROT (**b**).

Microscopic examination revealed marked neurodegeneration in the midbrain of rats injected with ROT. Treatment with CJ ameliorated the neuron loss as only a small number of deeply stained (damaged) neurons were observed. Rats treated with the juice alone represented normal brain structure (Figure 4a). The prolonged treatment with ROT resulted in a cell loss by 57% compared to control, while administration of CJ improved the cell survival by 41%. Surprisingly, treatment with cranberry juice alone slightly affected cells’ survival as a 15% decrease in cell number was observed compared to control animals (Figure 4b).

### 3.4. Postural Instability

**Figure 5:**
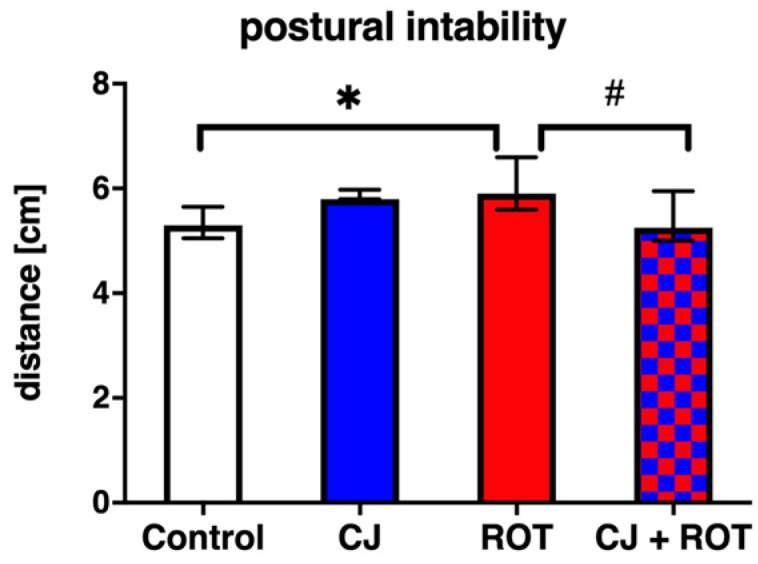
Effect of cranberry juice (CJ) treatment on postural instability in rotenone (ROT)-injected rats. Data are presented as median (bars) and interquartile range (whiskers) of eight rats per group and analyzed using Kruskal-Wallis followed by Dunn’s multiple comparisons test. * *p* < 0.05 vs. Control. # *p* < 0.05 vs. ROT.

The changes at the molecular and cellular levels correlated well with behavioral deficits. Animals injected with ROT exhibited 13% greater postural instability (statistically significant) than the control, measured as increased distance. The degree of postural impairment was 11% less in rats that received CJ and ROT in combination and similar to the control group (Figure 5).

## 4. Discussion

Although the PD prevalence has risen quickly worldwide, there is still a lack of disease-modifying therapy. Thus, the optimal management of the disease requires a multidisciplinary team approach that involves a growing number of recently developed non-pharmacological interventions [27]. Natural compounds with high biological activity and relatively low potential for side effects have gained special attention in this context [28]. Studies on dietary PPs suggest their beneficial role against PD, mainly attributed to antioxidant, anti-inflammatory, antiapoptotic, and autophagy modulation activities [29,30]. Treatment with ellagitannin-rich pomegranate has been demonstrated to protect against PD, which was manifested by improved motor and olfactory deficits, enhanced neuronal survival, and dopamine release correlating well with protection against oxidative damage and α-synuclein aggregation and maintenance of antiapoptotic potential at the control level in the midbrain [7,31]. Cranberry fruit containing high quantities of PPs, such as anthocyanins and F3O, also in the condensed form of PACs [9], represents the potential of neuroprotective activity as well. CJ treatment attenuates oxidative stress in several organs, with the most noticeable effect in the brain [25].

The pathological hallmark of PD is insoluble aggregates of the presynaptic protein-α-synuclein, deposited in LBs [32]. It has been demonstrated in several animal and cellular models that overexpression of this protein results in neurotoxicity as most soluble α-synuclein converts into amyloid fibrils [33]. The rate of α-synuclein synthesis and clearance maintains this protein at the proper level in the central nervous system, and failure in any of these mechanisms leads to its accumulation [34]. Thus, targeting the α-synuclein accumulation is a desirable option for neuroprotective intervention in PD [35]. Accumulation of α-synuclein has been demonstrated in the rats’ midbrains following subcutaneous administration of ROT [7] and ROT-treated human neuroblastoma-derived cells [36]. In this work, we have demonstrated for the first time that CJ treatment decreased α-synuclein expression in the midbrain of rats co-treated with CJ and ROT. Experimental evidence has shown that rich in PACs grape products significantly decreased the α-synuclein accumulation in the frontal cortex of transgenic mice with overexpression of the A53T-mutant human cells [37] and in ROT-treated human neuroblastoma-derived cells [36]. Oxidative stress is suggested to play a crucial role in PD pathogenesis. The abnormal form of α-synuclein interacts with transition metals or other components, promoting ROS production, and vice versa ROS may also potentiate the oligomerization of α-synuclein. Moreover, proteotoxicity and mitochondrial dysfunction have been widely demonstrated to be interdependent in neurodegeneration. As evident from studies with isolated mitochondria, cultured cells, and postmortem brain samples, the aggregated or oligomerized α-synuclein–or even its overexpressed monomers-cause mitochondrial damage. Conversely, ROS generated due to impairment of mitochondrial function may also potentiate the oligomerization of α-synuclein [38]. The rat model with neurotoxin ROT being administered chronically in relatively low-dose develops PD-like neurodegeneration due to sustained inhibition of complex I, related oxidative injury, and α-synuclein aggregation [6,7]. The catecholaldehyde hypothesis for the pathogenesis of PD imputes deleterious interactions between ROS-induced aldehydes, especially dopamine-derived 3,4-dihydroxyphenylacetaldehyde (DOPAL) and α-synuclein leading to oligomerization of the protein [39]. Our previous findings that CJ ameliorated lipid peroxidation, DNA damage and elevated activity of aldehyde dehydrogenase 2 (ALDH2) detoxifying DOPAL in the brain [25] support the protective effect of CJ treatment on ROT-induced α-synuclein accumulation.

Toxic products of lipid peroxidation have been demonstrated to induce apoptosis with activation of caspases -8, -9, and -3 and DNA fragmentation [40]. Mitochondrial complex I inhibitor-rotenone induces cell death by enhancing mitochondrial reactive oxygen species production. Rotenone-induced apoptosis was accompanied by an increased release of mitochondrial-related apoptotic proteins [41,42]. In the present study, we observed an increase in the Bax, cytochrome c, and cleaved caspase-9 expression in ROT-challenged rats. CJ treatment declined cleaved caspase-9 and Bax expression, while Bax and cytochrome c levels in the midbrains of rats given combined treatment with CJ and ROT were similar to the control values. Ma et al. (2018) demonstrated that PACs decreased rotenone-induced ROS production and elevated the level of active/cleaved caspase-9, therefore the enhancing survival of human neuroblastoma SH-SY5Y dopaminergic cells [43]. PACs increased also cell viability and reduced cell apoptosis in 1-methyl-4-phenyl-1,2,3,6-tetrahydropyridine (MPTP)-treated PC12 cells [44]. In rats, grape seeds PACs (GSP) effectively reduced pentylenetetrazole-induced hippocampal dysfunction and improved cognitive decline, in part, by suppressing caspase-3-mediated apoptosis [45]. PACs also protected neurons from cypermethrin-induced oxidative insult, decreasing ROS generation, relieving mitochondrial membrane potential loss, repairing nuclear morphology, and reducing cell apoptosis [46]. Grape seed PACs protected mice against ischemic stroke via attenuating neuronal apoptosis. GSP attenuated ER and mitochondrial stress-associated apoptosis by inhibiting glucose-regulating protein and caspase-12 [47]. GSPs also protected rats against iron overload-induced neuronal apoptosis by maintaining the metal balance, reducing oxidative stress, and regulating apoptotic gene expression [48].

In PD, a movement disorder develops caused by progressive loss of nigrostriatal DAergic neurons, and neuroprotective strategies aim to slow or stop the neurodegenerative processes. Epidemiological research has suggested that the intake of dietary PACs may reduce the risk of PD [49]. Administration of A-type procyanidins from cinnamon to MPTP-treated mice attenuated MPP+/MPTP-induced dopaminergic neuronal death and prevented the impairment of locomotor activity. The neuroprotective effects were mediated via the downregulation of the P38MAPK/P53/Bax signaling pathway and related decreases in oxidative stress, mitochondrial dysfunction, and apoptosis [50]. Chen et al. (2018) have demonstrated that PACs treatment ameliorated MPTP-induced bradykinesia that correlated with increased survival of DArgic neurons [44]. The authors have revealed that the neuroprotective activity of PACs was accompanied by the inhibition of ROS generation and modulation of c-Jun N-terminal kinase activation [44]. In our present study, CJ treatment protected against ROT-induced postural instability counteracting the neuronal loss in the midbrain of rats. 

Previously we have reported that the polyphenol-rich CJ with strong antioxidant activity controls oxidative stress in several organs with the most noticeable effect in the brain [25]. The present study provides experimental evidence for neuroprotective effects of CJ in rats. Importantly, we found that long-term administration of CJ alone in a relatively high dose may adversely influence cell survival in the midbrain. However, this effect was not accompanied by the upregulated expression of pro-apoptotic factors. Interestingly, as the expression of procaspase-9 was increased (Figure S1) it could be suggested that rats treated with CJ alone were deficient in their ability to cleave this protein. Recently, it has been demonstrated that when caspase-9 was inhibited by artemisinin from Artemisia carvifolia, it promoted autophagy to induce apoptosis via the caspase-8 activation [51]. Moreover, polyphenols have been reported to act as antioxidants or prooxidants depending on dose, duration of treatment, and physiological redox status [52]. In this context, pro-apoptotic and anti-proliferative effects are the most prominent for F3O and flavonols [53], which like (+)-catechin and (−)-epicatechin, can pass the BBB [54]. Given the potential pro-oxidant and pro-apoptotic capacity of PACs-rich plant products and related autophagy enhancing mechanisms, further investigation into their dose-dependent neuroprotective effects is necessary.

## 5. Conclusions

CJ slightly improved ROT-induced behavioral deficit by protecting from apoptosis and α-synuclein accumulation in the midbrain of rotenone-treated rats, demonstrating its neuroprotective efficacy for PD. These findings suggest that cranberry preparations may have a potential application in clinical practice or dietary guidelines for the prevention and/or adjunctive treatments of PD. However, taking into account the potentially unfavorable effect of long-term administration of juice in relatively high doses, a dose–dependence study should be performed to optimize the treatment.

## Figures and Tables

**Table 1:** Rat groups and treatments.

Group (n)	Treatment	
	1st–10th Day	11th–45th Day
Control (n = 11)	water (*i.g.*)	water (*i.g.*) + oleum (s.c.)
CJ ^1^ (n = 11)	CJ ^1^ (*i.g.*)	CJ ^1^ (*i.g.*) + oleum (s.c.)
ROT ^2^ (n = 11)	water (*i.g.*)	water (*i.g.*) + ROT ^2^ (s.c.)
CJ + ROT (n = 11)	CJ ^1^ (*i.g.*)	CJ ^1^ (*i.g.*) + ROT ^2^ (s.c.)

CJ, Cranberry juice, ROT, rotenon; *i.g*., intragastrically; s.c., subqutaneous; b.w., body weight ^1^ (500 mg/kg b.w./day), ^2^ (1.3 mg/kg b.w./day).

## Data Availability

Original data are available with the authors according to their contribution but not archived in databases elsewhere.

## Supplementary Materials

The following supporting information can be downloaded at: https://www.mdpi.com/article/10.3390/nu14102014/s1, Figure S1. Effect of cranberry juice (CJ) treatment on procaspase-9 expression.
