# Supplementary material for: Neuroprotective Effects of Cranberry Juice Treatment in a Rat Model of Parkinson’s Disease
Source: Nutrients. 2022 May 11;14(10):2014. doi: 10.3390/nu14102014 (PMC9144186; doi:10.3390/nu14102014)
Supplement: Supplementary file 1 [file nutrients-14-02014-s001.zip › nutrients-1693690-supplementary.pdf]

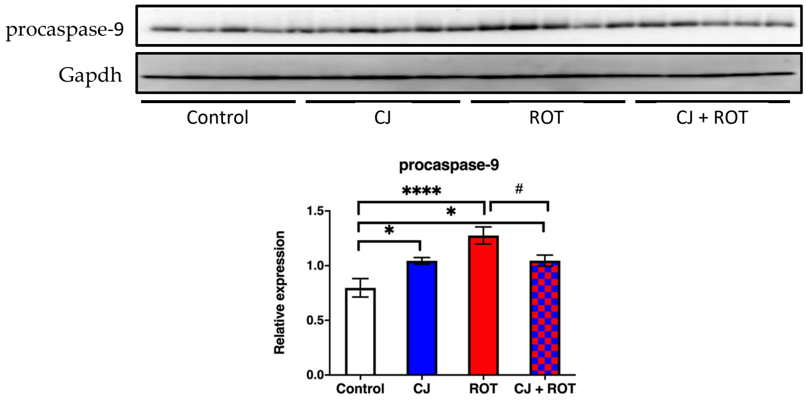

Figure S1. Effect of cranberry juice (CJ) treatment on procaspase-9 expression. Top panel shows a representative immunoblots. Graphs show relative expression ± SEM of procaspase-9 normalized to GAPDH in the midbrain of rotenone (ROT) injected rats (n = 8/group). Data were analyzed using one-way ANOVA followed by Fischer's LSD multiple comparisons test. * $p < 0.05$ vs. control; **** $p < 0.001$ vs. control; # $p < 0.05$ vs. ROT.
